# Oxidative Stress Modulators and Functional Foods

**DOI:** 10.3390/antiox10020191

**Published:** 2021-01-29

**Authors:** Junsei Taira

**Affiliations:** Department of Bioresources Technology, Okinawa College, National Institute of Technology, 905 Henoko, Okinawa, Nago 905-2192, Japan; taira@okinawa-ct.ac.jp

Many years of research have seen the investigation of natural antioxidants and dietary supplements as functional foods with the potential to prevent oxidative stress due to the scavenging of reactive oxygen species (ROS) and reactive nitrogen species (RNS). Recent studies have also revealed that cell signalling pathways, such as Nrf2-ARE, signalling with antioxidant protein (heme oxygenase-1, HO-1) expression, play significant roles in the cell’s survival response to avoid cell damage by the excessive production of ROS, RNS, or electrophiles. Therefore, natural products extending beyond the traditional antioxidant role are gaining a great deal of attention in functional foods, which can protect against various diseases related to oxidative stress. This Special Issue consists of 15 articles related to the antioxidant role of natural products, but also their ability to modulate oxidative stress and/or reverse disease both in vitro and in animal models. Additionally, the molecular mechanisms of these actions and the modulation of the signalling pathways in the redox system by natural products are included. 

Folic acid (FA) is known as a dietary supplement that can prevent neural tube defects (NTDs), involving the failure of neural tube (NT) closure in the developing embryo, especially spina bifida and anencephaly in the periconceptional period. Previous study indicated that moderate levels of nitric oxide (NO) and nitric oxide synthase (NOS) play a critical role in normal embryonic development. NO inhibits methionine synthase (MS), involving the interference transfer of the methyl group from the methyl donor, 5-methyl-tetrahydrofolate through the FA metabolite system (Folate pathway), to homocysteine during methionine production. Taira et al. [1] elucidated that FA can directly scavenge NO, suggesting that NO modulation, due to FA, may contribute to alleviation from failure in neural tube formation, causing the high level of NO production. 

Understanding the structure–activity relationships of antioxidants and their mechanisms of action is important for designing more potent antioxidants for potential use as therapeutic agents as well as preservatives. The kinetic studies of antioxidative action of ellagic acid (EA) under physiological conditions elucidated that the hydroxyl radical (•OH) with EA conforms to hydrogen atom transfer and radical adduct formation mechanisms, whereas the sequential proton loss electron transfer mechanism is responsible for the scavenging of the CCl_3_OO• radical, generating in the organism during the metabolism of CCl_4_. In addition, compared to trolox, EA was found to be more reactive toward •OH, but less reactive toward CCl_3_OO• in which their calculated rate constants are in very good agreement with the corresponding experimental values [2]. From another viewpoint of antioxidant mechanisms, the computational study for antioxidant mechanisms was carried out using total enthalpy values on the electronic effects of ortho-substituents in dendritic tri-phenolic antioxidants, comprising a common phenol moiety and two other phenol units with electron-donating or electron-withdrawing substituents. As the preferred antioxidant mechanism, in sequential proton loss electron transfer (SPLET) it was found that electron-donating groups, such as the OCH_3_ group, are useful for designing potent dendritic antioxidants, while the nitro and halogens do not add value to the radical scavenging antioxidant activity [3]. Furthermore, to predict the antioxidant potentiality in vivo, Boix et al. [4] proposed that the zebrafish model assay has the capability to predict in vivo protective activity or to determine their underlying mechanisms of action. This article showed a useful experimental system to evaluate the in vivo protective effects of different antioxidant compounds, based on the zebrafish embryo test under oxidative stress conditions using tert-butyl hydroperoxide, tetrachlorohydroquinone and lipopolysaccharide chemicals. This system was also applied to the study of the effects of well-known antioxidants, such as vitamin E, quercetin, and lipoic acid, and confirmed the zebrafish model as a useful in vivo tool to test the protective effects of antioxidant compounds.

Recent studies have revealed that cell signalling pathways, such as Nrf2-ARE signalling with HO-1 expression play significant roles to avoid cell damage causing oxidative-related various diseases. Taira et al. [5] focused on exploring the Nrf2 active compound and over five hundred various edible medicinal herbs were evaluated by a reporter assay, and the highest Nrf2 activity was found in the ethanol extract of *Peucedanum japonicum* leaves. The active compound in the extract was identical to pteryxin based on ^1^H, ^13^C-NMR spectra and liquid chromatography/time-of-fright/mass spectrometry (LC/TOF/MS). Pteryxin accumulated the transcription factor Nrf2 in the nucleus and resulted in the expression of the HO-1. This study also suggested that the electrophilicity, due to α,β-carbonyl and/or substituted acyl groups in the molecule, modulates the cysteine residue in Keap1 via the Michel reaction, at which point the Nrf2 is dissociated from the Keap1. Furthermore, the Nrf2 activator in bioresource reported the function in relation to disease. Osteoarthritis (OA) is a common joint degenerative disease induced by oxidative stress in chondrocytes. Kim et al. [6] demonstrated the inhibitory effects of cudratricusxanthone O (CTO), isolated from the *Maclura tricuspidata* Bureau (Moraceae), on the H_2_O_2_-induced damage of SW1353 chondrocytes. CTO induced HO-1 expression, involving the translocation of Nrf2 into the nucleus. Pretreatment with CTO in H_2_O_2_-treated cells regulated ROS production by inducing, expression of antioxidant enzymes (SOD, catalase, glutathione peroxidase (GSH-Px), glutathione reductase, and HO-1, and also prevented H_2_O_2_-induced apoptosis by regulating the expression of anti-apoptotic proteins, such as Bcl-2 and Bax. Environmental stress, involving oxidative stress, due to Ultraviolet (UV) and air pollutants contributing fine dust (FD) containing hazardous chemicals, induces triggering allergic reactions and inflammation of the skin, which lead to thickening of the epidermis, discoloration, skin wrinkling, loss of elasticity and skin-cell growth retardation [7,8]. Fernando et al. [7] reported that a low molecular weight fucoidan fraction (SHC4, 60 kDa, with 37.43% fucose and 28.01% sulfate), isolated from *Sargassum horneri*, reduced intracellular ROS levels and increased the cell viability on UVB (280–320 nm) exposed HaCaT keratinocytes and inhibited UVB-induced apoptotic body formation, sub-G1 accumulation of cells through the mitochondria-mediated pathway. The UVB protective effect of SHC4 was facilitated by enhancing intracellular antioxidant defence via Nrf2-HO-1 signalling. The FD in air pollutants also produced the ROS in human HaCaT keratinocytes. (–)-loliolide (HTT), isolated from *Sargassum horneri*, has the potential to increase cell viability by reducing the ROS production in FD-stimulated keratinocytes, involving the mitochondria-mediated apoptosis pathway. HTT suppressed FD-stimulated DNA damage and the formation of apoptotic bodies, and it reduced the population of cells in the sub-G1 apoptosis phase. The cytoprotective effects of the HTT against FD-stimulated oxidative damage is mediated through squaring the Nrf2-HO-1 pathway involved in increasing HO-1 and NAD(P)H dehydrogenase (quinone) 1 in the cytosol [8].

In this Special Issue, the approach to find new biological activities of antioxidants in relation to various diseases and the different bioactivities, due to habitat-derived conditions, were reported by the following: (1) Type 2-diabetes mellitus (T2-DM) is caused by hyperglycaemic abnormalities in controlling blood glucose and insulin resistance. This article showed the mechanism of ameliorative effects due to quamoclit angulata (QA) on diabetes. QA supplementation (5 or 10 mg/kg/day) for 12 weeks reduced homeostasis model assessment insulin resistance, kidney malfunction, and glomerular hypertrophy in T2-DM. Moreover, the QA treatment significantly attenuated renal NLRP3 inflammasome-dependent hyper-inflammation and the consequential renal damage caused by oxidative stress, apoptosis, and fibrosis in T2-DM [9]. (2) In a similar model animal experiment, the high sugar-fat (HSF) diet induced obesity, insulin resistance, cardiac dysfunction, and oxidative damage. When tomato-oleoresin supplementation (containing 10 mg lycopene/kg body weight (BW) per day) was given orally every morning for a 10-week period, the insulin resistance, cardiac remodelling, and dysfunction were improved by regulating the β-adrenergic response. [10]. (3) The antioxidant properties of epigallocatechin-gallate (EGCG), a green tea compound, have been already studied in various diseases. To improve the bioavailability of EGCG, this article demonstrated the result of the comparative effect of liposomal EGCG (L-EGCG) with EGCG solution in experimental DM induced by streptozotocin in rats. L-EGCG indicated a better efficiency regarding the improvement of oxidative stress parameters for malondialdehyde (MDA), NO, and total oxidative status; antioxidant status for total antioxidant capacity of plasma, thiols, and catalase and matrix-metalloproteinase-2 were also significantly reduced in the L-EGCG-treated group, compared with the EGCG group [11]. (4) Myricetin is present in many natural foods with various biological activities, such as anti-oxidative and anti-cancer activities. Benzo[a]pyrene (B[a]P), a group 1 carcinogen, induces mutagenic DNA adducts. B[a]P is metabolized by phase I enzymes, cytochrome P450 (CYP), and CYP1A1 also produces the metabolites conjugated with the DNA-BPDE (B[a]P-7,8-dihydrodiol-9,10-epoxide) adduct and 8-hydroxy-2’-deoxyguanosine formation. This article showed that myricetin reduces B[a]P-induced toxicity by inhibiting those metabolites by the reduction of the B[a]P metabolism via reduced CYP1A1 expression, and the elimination of B[a]P metabolites via enhanced GST expression [12]. (5) The variations in the phenolic profile for 21 compounds and bioactivities for antioxidant and antimutagenic activities between bilberry and lingonberry leaves different from three locations due to different altitude, solar exposure and temperature range were investigated. As a result, flavonols, hydroxycinnamic acids, and anthocyanins, due to habitat-derived conditions, could be clearly distinguished in these species [13].

As a large molecule antioxidant, a novel pectic polysaccharide, SAZMP4 (M.W, 28.94 kDa), mainly containing 1,4-linked galacturonic acid (GalA, 93.48%), with side chains of various neutral sugars, such as rhamnose, arabinose was isolated from Jujube pomace and the structure was determined by GC, FI-IR, GC-MS, NMR for molecule analysis, and SEM (scanning electron microscope) and AFM (atomic force microscope) for molecular morphological analysis. In addition, the antioxidant activity of SAZMP4 against H_2_O_2_-induced oxidative stress in Caco-2 cells demonstrated SOD activity and GSH-Px, MDA. Additionally, a better water retention capacity and the thermal stability of SAZMP4 indicated a potential application in the food industry as an additive [14].

The review article in this Special Issue discussed cellular and genetic factors that increase oxidative stress in Parkinson’s disease (PD). PD is a neurodegenerative disorder caused by the depletion of dopaminergic neurons in the basal ganglia, the movement centre of the brain. The accumulation of oxidative stress-induced neuronal damage, due to the increased production of ROS or impaired intracellular antioxidant defences, invariably occurs at the cellular levels. The dopaminergic prodrugs and agonists can alleviate some of the symptoms of PD, but they could not be completely prohibited by the progression of PD pathology. The progress of PD takes a long time for the neurodegenerative process; therefore, the authors proposed that strategies to prevent or delay PD pathology may be well suited to lifestyle changes, such as dietary modification with antioxidant-rich foods, including vitamin C, vitamin E, carotenoids, selenium, and polyphenols [15]. 
